# Moving beyond a ‘one-size-fits-all’ rationale in global mental health: prospects of a precision psychology paradigm

**DOI:** 10.1017/S2045796021000500

**Published:** 2021-10-11

**Authors:** Marianna Purgato, Rakesh Singh, Ceren Acarturk, Pim Cuijpers

**Affiliations:** 1 Department of Neurosciences, Biomedicine and Movement Sciences, WHO Collaborating Centre for Research and Training in Mental Health and Service Evaluation, University of Verona, Verona, Italy; 2 Cochrane Global Mental Health, University of Verona, Verona, Italy; 3 Department of Public Health, Independent Mental Health Researcher, Visiting Faculty, KIST Medical College, Kathmandu, Nepal; 4 Department of Psychology, College of Social Sciences and Humanities, Koc University, Istanbul, Turkey; 5 Department of Clinical, Neuro, and Developmental Psychology, Amsterdam Public Health Institute, Vrije Universiteit, Amsterdam, Netherlands; 6 WHO Collaborating Centre for Research and Dissemination of Psychological Interventions, Vrije Universiteit, Amsterdam, Netherlands

**Keywords:** Global mental health, humanitarian settings, low- and middle-income countries, precision psychology

## Abstract

Research on the effectiveness of mental health and psychosocial support interventions for common mental disorders in global mental health provides controversial results. These results are based on mean values for different groups, often without due consideration of individual-level characteristics and contextual factors. Against this background, and based on the recent development of a precision theoretical framework in clinical psychology, which is calling for a renewed perspective on the development and implementation of trial designs, we propose to develop a precision psychology paradigm in global mental health, with emphasis not only on individual clinical and socio-demographic data, but also on the social determinants of mental health. A precision psychology paradigm would require a coordinated action of academics, stakeholders and humanitarian workers in planning a global mental health research agenda, including the design of trials aimed at reliably approximate prediction of intervention response at individual level.

## Introduction

UN estimates suggest that in 2021 nearly 238 million people in more than 40 countries around the world will need humanitarian assistance and protection resulting from conflict or disaster. Nearly 69 million people worldwide have been forcibly displaced by violence and conflict, the highest number since World War II (UNOCHA, 2019, 2021). Political instability and natural disasters disproportionally affect populations living in low- and middle-income countries (LMICs), where extreme chronic poverty (less than 1,90Int.$ per day) (World Bank, 2018) causes severe deprivation of basic human needs, including food, water, sanitation, health and education (Guha-Sapir *et al*., 2014). In 2019, the United Nations (UN) verified over 25 000 grave violations against children including killing and maiming, recruitment of children as soldiers, sexual violence and abductions, as well as 927 attacks on schools and hospitals (UNOCHA, 2021).

A humanitarian crisis is ‘an event or series of events that represents a critical threat to the health, safety, security or well-being of a community or other large group of people, usually over a wide area’ (Sphere Association, 2018). Humanitarian settings involve a broad range of situations, including natural disasters, armed conflicts, slow- and rapid-onset events, rural and urban environments (e.g., chronic extreme poverty) and complex political emergencies (Tol *et al*., 2011).

The definition of humanitarian setting reported above may refer to any phase of the emergency or recovery process, in consideration of the fact that psychological effects of trauma and adversities endure long after ‘the last shot is fired’ (Comtesse *et al*., 2019).

In addition, the coronavirus disease-2019 (COVID-19) pandemic has brought with it the physical sequelae of the viral infection and increased levels of poverty, socioeconomic insecurity and mental health problems. Measures implemented to contain the spread of COVID-19 have increased or amplified humanitarian access constraints, hunger and geopolitical tensions that were on the rise even before the pandemic. These restrictions have also triggered delays, additional costs and the partial suspension of humanitarian activities (UNOCHA, 2021). For children, adolescents and youths school closure weakened friendship and social support networks (Singh and Subedi, 2020; Singh *et al*., 2021), protection from risk-taking behaviours, exploitative labour and child abuse.

The mental health and psychosocial consequences of the COVID-19 pandemic, added to the well-known challenges of living in humanitarian settings, increased levels of acute distress and risk of developing mental disorders and co-morbidities (Kola *et al*., 2021).

Research examining the effects of war and trauma on children, adolescents and adults in humanitarian settings in LMIC showed significant levels of psychological difficulties and psychiatric problems after exposure to conflict. However, prevalence data are inconsistent and likely depend on the nature of the trauma, the duration of exposure, diagnostic criteria used and differences in methodological factors as sampling and assessment tools (e.g., self-report *versus* clinician administered) and cultural discrepancies (Attanayake *et al*., 2009; Kar, 2009). Psychological problems like generalised anxiety, posttraumatic stress disorder (PTSD) and depression are prevalent after exposing individuals to war and terrorism. A study among 3370 adolescents exposed to traumatic events in ten LMICs found that 28.5% of adolescents endorsed two to three DSM-5 PTSD criteria symptoms, while the rates of adolescents with symptoms from all four DSM-5 criteria for PTSD ranged from 6.2 to 15.3% (Stupar *et al*., 2021). The most frequently reported traumatic events were being exposed to death of a close person, witnessing violence other than domestic, experiencing a natural disaster and witnessing violent death or serious injury of a close person (Stupar *et al*., 2021).

A recent systematic review and meta-analysis across 129 studies by Charlson and colleagues estimated the prevalence of mental disorders in conflict settings, considering a 10-year frame (Charlson *et al*., 2019). Prevalence of mental disorders (PTSD, depression and mixed anxiety disorders) was 22.1% [95% confidence interval (CI) 18.8–25.7] at any point in time in the conflict-affected populations considered. For children, estimates highlighted higher prevalence of anxiety and PTSD compared with depression and consistently mean prevalence of PTSD declined in the older age groups (Charlson *et al*., 2019). A similar trend of increased prevalence of common mental disorders was identified by systematic reviews and meta-analyses focused on asylum seekers and refugees resettled either in LMICs or in high-income countries (HICs) (Blackmore *et al*., 2020a, 2020b).

Despite the reported prevalence rates, however, mental health is still no priority in most LMICs. For example, the median number of psychiatrists per 100 000 populations is only 0.1 in LMICs as compared to 11.9 in HICs (WHO, 2017). Furthermore, mental health care delivery at the community level depends almost exclusively on non-specialised health workers, despite the limited training on mental health care in primary health workers. This lack of training is even more affected by their sudden and frequent transfer to other health facilities, which leads to hindrances in providing mental care to the beneficiaries for long run (Luitel *et al*., 2015; Upadhaya *et al*., 2017; Mishra *et al*., 2018). For example, in Nepal the proportion of total annual health budget has been less than 1% for mental health since many years, with mental health services being concentrated in major cities. The number of psychiatrists, psychologists and psychiatric beds has been 0.22, 0.06 and 1.5 per 100 000 populations, respectively (Singh *et al*., 2020). Henceforth, these various hindering factors could further pose a serious threat to mental health wellbeing among people in LMICs when Mental Health and Psychosocial Support (MHPSS) interventions are run based on a ‘trial and error approach’.

Unfortunately, wars and humanitarian crises continue around the world, and it is necessary to keep researching their consequences on mental health and to identify safe and effective evidence-based interventions to reduce psychological suffering.

Despite the growing interest over the last decade for testing MHPSS interventions, results from systematic reviews and meta-analyses of randomised controlled trials (RCTs) are heterogeneous and controversial, especially for common mental disorders (e.g., PTSD, depression and anxiety symptoms). The current research paradigm is mainly focused on testing interventions' effectiveness using RCT designs, which may show favourable as well as unfavourable effects depending on the study timeline for outcome evaluation and contextual and individual factors (Ertl and Neuner, 2014; Barbui *et al*., 2020). A new paradigm may be developed to account for individual and contextual factors (i.e., social determinants of mental health) by considering subgroups of individuals with specific clinical and socio-demographic characteristics to identify predictors of intervention response (Kravic, 2020; Furukawa *et al*., 2021).

This editorial first summarises the evidence available on MHPSS interventions for children, adolescents and adults living in humanitarian settings in LMICs. Then, it describes the precision medicine approach reporting the example of general medical fields in which a precision approach was developed and tested. Finally, we argue about a precision paradigm applied to clinical psychology and particularly to global mental health.

## Effectiveness trials on mental health and psychosocial support interventions: the current paradigm

For the management of mental health conditions, a number of MHPSS interventions have been developed and formally tested for effectiveness over the last decades. A Cochrane systematic review of psychological therapies in humanitarian settings in LMICs identified 33 RCTs with 3523 participants. For adults, meta-analyses showed that psychological therapies substantially reduced symptoms of PTSD, depression and anxiety at study endpoint (0–4 weeks after the intervention ended), consistently with a review focused on MHPSS interventions in emergency settings in LMICs (Bangpan *et al*., 2019), but the intervention effect and the number of participants assessed gradually decreased over time. In children and adolescents between 5 and 18 years of age, four studies of very low quality found no significant beneficial effects of interventions over waiting list in reducing PTSD symptoms. No data were available in these studies for depression and anxiety (Purgato *et al*., 2018a, 2018b). Similarly, a Cochrane systematic review of psychological and social prevention interventions in humanitarian settings in LMICs highlighted the paucity of randomised evidence properly evaluating prevention interventions (i.e., incidence) and confirmed no beneficial effects of interventions over waiting list in reducing symptoms of PTSD, depression and anxiety in children. For adults, a positive effect of interventions in reducing depressive and anxiety symptoms at immediate post-intervention was detected, with no data on PTSD (Papola *et al*., 2020). Recently, two large RCTs evaluated a new psychological intervention called Self-Help Plus (SH+) and developed by the WHO, with the aim of preventing the onset of mental disorders in asylum seekers and refugees. These trials identified a beneficial effect of SH+ in preventing the frequency of mental disorders at post-intervention (Purgato *et al*., 2021) and at 6-month follow-up (Acarturk *et al*., in press).

In an attempt to move a step beyond effectiveness evaluation, a systematic review on focused psychosocial interventions used individual participant data (IPD) meta-analytic techniques to delineate socio-demographic profiles of children who might respond better to the allocated interventions. The IPD meta-analysis, conducted on 11 RCTs with 3143 children in humanitarian settings in LMICs (mainly in war contexts), found reduced PTSD and distress symptoms in specific subgroups of children only, e.g., older children of 15–18 years, or those not displaced, or those living in smaller households with less than six people (Purgato *et al*., 2018b, 2020a, 2020b). The IPD meta-analysis identified socio-demographic variables that might impact interventions' effect, however it was restricted to focused psychosocial support interventions without collecting information for psychotherapy or social interventions and included mixed samples of children (i.e., children with increased level of distress together with those with a potential psychiatric diagnosis). Moreover, the moderators identified by the IPD meta-analysis were not subsequently tested in formal RCTs.

According to this premise, we are presently unable to predict which subgroup of individuals might benefit most from which type of MHPSS intervention, forcing a ‘trial and error’ approach that: (1) might generate harm; (2) is almost certainly implicated in high rates of intervention dropouts; (3) causes delays in recovery and/or benefits in the short-term only; (4) and generates ethical concerns. These issues are problematic in low-resource contexts, where providing the right intervention to the right individual at right time may be a matter of survival, as many people are exposed to multiple traumatic events, and there is intense scarcity of human resources in mental health.

Although it should be recognised that the current paradigm focused on effectiveness trials providing mean values at group level has been crucially important over the last decade for the development and implementation of large RCTs on MHPSS interventions' effect, progress should now be made towards the identification and testing of predictors of interventions' response, to generate evidence for matching interventions with subgroups of individuals that might respond optimally.

This could, in turn, help minimise the inequity gap between HICs and LMICs in terms of provision of safe and effective mental health interventions.

## Precision medicine

Taking into account individual clinical data, biological susceptibility and social and environmental factors, precision medicine has a potential to tackle the challenges reported above, by generating a match between subgroups of individuals and the most appropriate available intervention (Cuijpers *et al*., 2012, 2016, 2019; Cuijpers and Christensen, 2017; Cuijpers and Beekman, 2018). Precision medicine aims at predicting which treatment option may work better for a particular disease in a specific group of people. Although used often synonymously with ‘personalised medicine’, the label ‘precision medicine’ has been recently adopted as some may misinterpret the word ‘personalised’ to imply treatments developed uniquely for each individual, while the term ‘precision’ implies the identification of which approach is best for a specific subgroup of individuals sharing specific characteristics. The concept of precision medicine dates back from Hippocrates in 400 BC to Claude Bernard, a physiologist in the 19th century. Archibald Garrod, who talked about the importance of individual ‘chemical differences’ in an article of 1902, is considered the father of precision medicine (Perlman and Govindaraju, 2016). He postulated that ‘no two individuals of a species are absolutely identical in bodily structure neither are their chemical processes carried out on exactly the same lines’ (Garrod, 1902). Being a physician, he emphasised the importance of clinical medicine and that is ‘in the ward rather than in the laboratory that the importance of inborn factors is to be appreciated […] and tricks of gesture and action’ (Garrod, 1902, 1909, 1931).

Up to now, precision medicine has been applied to different medical fields in Western HICs, offering new avenues for the amelioration of diseases. In 2015, a Precision Medicine Initiative was promoted by Obama in the United States to accelerate progress and research towards curing diseases and providing access to personalised information (Collins and Varmus, 2015). The goal of this initiative was to improve health by tailoring the prevention and treatment interventions of health conditions to genetic, environmental and lifestyle differences among individuals. Because of the progress in sequencing the genome of cancer cells and in identifying mutations that are ‘drivers’ of malignancy, oncology was mentioned amongst the main areas of interest, as in the last 20 years different trial designs adopting a precision medicine approach were developed and tested in oncology (Manrai *et al*., 2016, 2018).

Of note, in psychiatry a precision paradigm has already been conceptualised, with emphasis on the need of accounting for the difficulties of translating genetic information into diagnosis and clinical interventions, as diagnoses are mainly based on clinical evaluations, depending on the clinicians' judgement and experience. Similarly, the subsequent prescription phase typically follows a ‘trial and error’ approach (Vieta, 2015). Nevertheless, research efforts have been directed to designing a new roadmap for precision psychiatry, aiming to overcome these barriers by stratifying disease processes, identifying predictors of treatment outcome and using neuroimaging and other biomarkers to evaluate the role and mechanisms of action of mental health interventions (Krystal *et al*., 2020; Williams *et al*., 2011). The International Study to Predict Optimized Treatment for Depression (iSPOT-D) is an example of a large RCT (*n* = 2016) aimed at identifying a number of predictors and moderators of treatment outcome in adult participants with major depressive disorder and to develop and test through a replication study a model to incorporate the effects of multiple baseline predictors or moderators on antidepressant response (escitalopram, sertraline or venlafaxine-extended release) (Williams *et al*., 2011). iSPOT-D represents an important step towards precision psychiatry, as it is hypothesised that baseline clinical data (e.g., symptom severity, psychological characteristics, cognitive functioning and physical parameters) may act as potential markers for treatment response.

## Precision psychology in global mental health: identifying and testing predictors of intervention response through a coordinated approach

Accurate prediction of which persons will benefit the most from which intervention would be possible even in the field of clinical psychology, where a theoretical framework is being developed for mental health conditions as depression (Huibers *et al*., 2021).

In a systematic review and meta-analysis aimed at assessing personalised treatments for adult depression, Cuijpers *et al*. collected 41 RCTs focused on specific population groups with 2741 participants, which were allocated to six different intervention strategies (head-to-head comparisons) based on their specific characteristics. Target groups were defined according to the availability of clinical trials focused on them and considering socio-demographic characteristics (e.g., older adults or minority groups), clinical characteristics (e.g., type of depression) and comorbid clinical conditions (e.g., physical illness). In total, authors identified 27 clinical and socio-demographic characteristics of participants as potential moderators of intervention's effect. However, only for a few set of characteristics the statistical power was sufficient to show a clinically relevant effect size. Moreover, there was high risk of bias in the included studies, and only a selected number of potentially relevant moderators were examined (Cuijpers *et al*., 2016). For example, CBT was found to be more effective than other therapies in older adults, in patients with comorbid addictive disorders and in university students, while for the other therapies there was insufficient statistical power to show that one therapy was more effective than another therapy.

Even though the results highlighted the need of using complementary strategies for developing a proper precision approach in psychology, the review approximated its application, offering an example that incorporated individual level characteristics in the choice of the intervention strategy.

In the area of global mental health, a precision psychology approach has never been conceptualised, developed and validated. Currently, the majority of clinical trials in LMICs are designed to assess the effectiveness of a target MHPSS intervention against a control condition – often no treatment or waiting list − with a focus on short-term outcomes (mainly immediate post-intervention) (Purgato *et al*., 2019). Little evidence exists on the mechanisms of action of interventions, for example considering specific interventions' ingredients or the mediation role of target variables to predict *how* a MHPSS intervention works. Secondly, trials are focused on mean-group differences, informing that a given characteristic was ‘on average’ different between groups, but without assessing variability across individual participants, making it impossible to predict *who* might benefit most from an intervention. In this way, even when an intervention is proven to be effective in reducing psychological symptoms, the evaluation refers to mean values of clinically heterogeneous groups of individuals. Thirdly, evidence does not always incorporate environmental and social variables to predict *under what set of circumstances* a specific intervention is optimal in terms of efficacy and safety. The social determinants of mental disorders are the social and economic conditions that have a direct influence on the prevalence and severity of mental disorders in individuals across the life course (Patel *et al*., 2018). There is growing global evidence that mental disorders in populations are strongly socially determined, especially in sensitive periods as childhood and adolescence, with the mediation of biological factors (Lund *et al*., 2018). In terms of theoretical frameworks on the relation between social determinants and mental health, Lund *et al*. recently developed a new conceptual framework that summarised the major social determinants of mental health and linked them with the Sustainable Development Goals. This framework – adopted by the *Lancet Commission of Global Mental Health and Sustainable Development* (Patel *et al*., 2018) – identified the following domains of the social determinants of mental disorders: demographic, economic, neighbourhood, environmental events and social and culture domains. In each domain, distal and proximal determinants were identified to impact on mental disorders, mediated by family-level and biological variables. This framework highlighted the importance of an ecological approach and the complex multidimensional way in which social determinants interact with key genetic determinants to affect mental disorders, and may inform the development and implementation of psychological interventions (Lund *et al*., 2018).

For example, adverse social and economic circumstances that are frequently experienced in humanitarian settings in LMICs, like poverty, income inequality, trauma exposure, interpersonal and collective violence and forced migration, are key determinants of mental disorders, and may impact individual symptoms and functioning over time (Miller *et al*., 2021).

A recent study aimed at delineating trajectories of psychological symptoms and resilience in 597 children exposed to multiple traumatic events in Burundi, Indonesia, Nepal and Sri Lanka, identified a positive association between the number of traumatic event types and psychological symptoms of PTSD, depression and anxiety (Purgato *et al*., 2020a, 2020b). In particular, time and trauma were the factors exerting most influence on trends over time, and the healing effect of time was strongly contextually determined. In a precision psychology paradigm for global mental health, the integration of the social determinants of mental health in RCT designs should be prioritised and considered at an individual level, including contextual factors, e.g., the nature of trauma, the time since trauma exposure and the amount and types of traumatic events. In turn, these factors may be integrated with functional and structural neuroimaging information, that is not always easily accessible in LMICs, and might be less critical in guiding the intervention choice according to recent claims of shifting the attention from the consideration of mental health problems as mostly brain disorders (Ioannidis, 2019).

Fourth, RCTs should include head-to-head comparisons between different MHPSS interventions (Cuijpers, 2016), for example including low resource psychosocial interventions *versus* more complex and resource intensive psychotherapeutic interventions in homogeneous groups (Turrini *et al*., 2021). Additionally, longitudinal naturalistic designs might track the psychological outcomes and resource pathways, accounting for the contextual factors from proximal to distal determinants, regardless of the administration of the intervention.

We are aware of the theoretical and practical complexities implied in the development of a precision psychology paradigm in global mental health, but new research methodologies and technologies are available. IPD and network meta-analytic techniques might be applied to all MHPSS interventions for developing large clinical datasets with up-to-date individual-level sociodemographic, clinical, biological and context-related information. The efficacy and safety of different MHPSS interventions, compared each other (head-to-head) and against inactive controls (e.g., waiting list, no treatment, care as usual) might this way be studied in relation to available individual-level information, following a precision psychology approach. Machine learning algorithms may represent an additional tool for generating visual representations of selected MHPSS intervention options and to create evidence-based matches between subgroups of individuals with a psychological condition (e.g., PTSD, depression, anxiety) and the most appropriate MHPSS intervention.

However, IPD meta-analyses typically collect information on very few individual-level variables, as the primary studies were designed to respond to specific research questions. Therefore, in order to move beyond a ‘one-size-fits-all’ rationale, we need a new generation of RCTs that use a common set of individual-level measures to be used as moderators of response. This implies identifying the moderators that might potentially impact the intervention's effect first and verifying whether the inclusion of these in RCTs indeed results in better outcomes for a selected group of individuals. Qualitative research involving end-users, key informants and stakeholders might also be useful as a preparatory phase of RCTs, to properly understand the peculiarity of specific implementation contexts (e.g., humanitarian settings), barriers, facilitators and challenges.

This process will hopefully result in a comprehensive global evidence base for MHPSS interventions open to all researchers in global mental health, with trials being easily compared and combined and no longer considered as separate entities of data collection.
